# Helmet CPAP to treat hypoxic pneumonia outside the ICU: an observational study during the COVID-19 outbreak

**DOI:** 10.1186/s13054-021-03502-y

**Published:** 2021-02-24

**Authors:** Andrea Coppadoro, Annalisa Benini, Robert Fruscio, Luisa Verga, Paolo Mazzola, Giuseppe Bellelli, Marco Carbone, Giacomo Mulinacci, Alessandro Soria, Beatrice Noè, Eduardo Beck, Riccardo Di Sciacca, Davide Ippolito, Giuseppe Citerio, Maria Grazia Valsecchi, Andrea Biondi, Alberto Pesci, Paolo Bonfanti, Davide Gaudesi, Giacomo Bellani, Giuseppe Foti

**Affiliations:** 1 ASST Monza, San Gerardo Hospital, Monza, Italy; 2 Department of Medicine and Surgery, University of Milan-Bicocca, Via Cadore 48, Monza, MB Italy; 3 ASST Monza, Desio Hospital, Desio, Italy

**Keywords:** Helmet continuous positive airways pressure CPAP, Noninvasive ventilation, Covid-19, Positive end expiratory pressure PEEP, Coronavirus pneumonia

## Abstract

**Background:**

Respiratory failure due to COVID-19 pneumonia is associated with high mortality and may overwhelm health care systems, due to the surge of patients requiring advanced respiratory support. Shortage of intensive care unit (ICU) beds required many patients to be treated outside the ICU despite severe gas exchange impairment. Helmet is an effective interface to provide continuous positive airway pressure (CPAP) noninvasively. We report data about the usefulness of helmet CPAP during pandemic, either as treatment, a bridge to intubation or a rescue therapy for patients with care limitations (DNI).

**Methods:**

In this observational study we collected data regarding patients failing standard oxygen therapy (i.e., non-rebreathing mask) due to COVID-19 pneumonia treated with a free flow helmet CPAP system. Patients’ data were recorded before, at initiation of CPAP treatment and once a day, thereafter. CPAP failure was defined as a composite outcome of intubation or death.

**Results:**

A total of 306 patients were included; 42% were deemed as DNI. Helmet CPAP treatment was successful in 69% of the full treatment and 28% of the DNI patients (*P* < 0.001). With helmet CPAP, PaO_2_/FiO_2_ ratio doubled from about 100 to 200 mmHg (*P* < 0.001); respiratory rate decreased from 28 [22–32] to 24 [20–29] breaths per minute, *P* < 0.001). C-reactive protein, time to oxygen mask failure, age, PaO_2_/FiO_2_ during CPAP, number of comorbidities were independently associated with CPAP failure. Helmet CPAP was maintained for 6 [3–9] days, almost continuously during the first two days. None of the full treatment patients died before intubation in the wards.

**Conclusions:**

Helmet CPAP treatment is feasible for several days outside the ICU, despite persistent impairment in gas exchange. It was used, without escalating to intubation, in the majority of full treatment patients after standard oxygen therapy failed. DNI patients could benefit from helmet CPAP as rescue therapy to improve survival.

*Trial Registration*: NCT04424992

## Background

In the early months of 2020 a massive COVID-19 pneumonia outbreak hit Italy. During the pandemic, an overwhelming number of patients suffering from hypoxemic respiratory failure presented to hospitals’ emergency rooms, burdening the health system to an unexpected extent. To face such a number of critically ill patients, intensive care beds were more than doubled, with an occupancy close to 100% [1].

One of the effective treatments for respiratory failure, particularly if applied early and in less severe patients, is noninvasive ventilation [2, 3]. As an example, continuous positive airway pressure (CPAP) delivered noninvasively by helmet proved superior to non-rebreathing oxygen mask in community-acquired pneumonia [4, 5]. The optimal treatment of COVID-19 pneumonia is still under debate, and some experts believe that providing a moderate level (< 10 cmH_2_O) of Positive End-Expiratory Pressure (PEEP) can match patient’s need during the first phase of the disease, albeit this must be balanced with the potential risk of delayed intubation [6–8], a topic on which a vigorous debate is ongoing [9].

The rationale for using helmet CPAP is that it is effective for treatment of respiratory failure, and presents many advantages as compared to noninvasive ventilation mask interface [10, 11]. In fact, it is generally well tolerated, air leaks are rarely an issue and it is associated with low pressure ulcer complications, particularly for prolonged therapy [12]. When treating COVID-19 affected patients, the use of helmets might bear the additional advantage of reducing virus environment contamination [13, 14]. Recent reports suggest that helmet CPAP can be effective for COVID-19 treatment [15–17], possibly combined with prone or lateral position [18–20]. In a multicenter cohort study, Aliberti et al. showed that helmet CPAP significantly increased PaO2/FiO2 from oxygen administration alone, but that treatment failure was frequent, leading to intubation and mechanical ventilation of 44% of the patients [21].

Early recognition of patients at high risk of treatment failure is crucial, both for individual treatment and resources allocation. We present the outcomes associated with treatment of respiratory failure with helmet CPAP during the COVID-19 outbreak in our hospital consortium, an approach which has been widely applied in Northern Italy. The primary aim of the present study is to describe the effects of helmet CPAP treatment during the COVID-19 pandemic, identifying early predictors of CPAP failure. Secondary endpoints were the description of patients’ outcomes based on the presence of ceiling of care.

## Methods

This is a retrospective observational cohort study of COVID-19 patients treated with helmet CPAP from March 3 to April 3, 2020. Data were collected in a local online registry as part of the STORM study (Spallanzani Institute Approval Number 84/2020; NCT04424992). Patients’ consent was waived.

### Organizational aspects

The study was conducted in the two hospitals of the ASST Monza (San Gerardo Hospital and Desio Hospital), which include a total of approximately 1200 beds, with about 35 ICU beds before COVID-19; ICU beds were increased up to one hundred during the surge. Several medical and surgical wards were converted in COVID-19 wards, capable of applying noninvasive CPAP. Ward teams were composed by physicians and nurses from several disciplines.

Our medical emergency team (MET) is composed by an intensivist, a resident and a critical care nurse. During the pandemic, the MET was doubled during the surge and evaluated critical patients defining treatment limitations (i.e., do not intubate order, DNI) together with the floor physician and the patient. A protocol was instituted for the use of helmet CPAP in COVID-19 patients who failed standard oxygen therapy (Additional File 1: Fig. S1); failure of standard oxygen therapy was defined as one of the following during spontaneous breathing in non-rebreathing oxygen mask: SpO_2_ < 93%, respiratory rate > 24 Breaths Per Minute (BPM), pCO_2_ < 35 mmHg, thoraco-abdominal asynchronies.

### CPAP delivery system

Our free-flow CPAP system (Additional File 1: Fig. S2) relied on a Venturi flow generator connected to the oxygen wall port; a value of 60 L/min or above was the target for the fresh gas mixture flowing into the helmet [22]. FiO_2_ was verified by a FiO_2_ meter and adjusted based on oxygen saturation, while PEEP (i.e., CPAP level) within the helmet was maintained by an adjustable mechanical valve. Helmet CPAP was managed by floor physicians and nurses; the most severe patients were referred directly to the MET and managed on the hospital floor until intubation was required, while the others were treated by non-intensivist physician and screened once a day by the MET for possible ICU admission. Intravenous sedation was not used in the wards during helmet CPAP treatment, except for palliative care of terminal patients; some patients might have received oral benzodiazepines for relief of anxiety.

### Patients and data collection

Inclusion criteria were: respiratory failure treated with helmet following standard oxygen therapy; and a positive Sars-CoV-2 nasopharyngeal swabs (real time polymerase chain reaction). Exclusion criteria were: futility of medical treatment due to expected short term death independent of COVID-19 pneumonia.

The local online registry was developed with REDCap cloud 1.5 (Phase Inc). We collected data regarding past history and frailty [23]; date of symptoms onset, hospital admission and helmet CPAP start (day 1); blood gas analysis before and after (normally between one and two hours) helmet CPAP start; data regarding the use of helmet CPAP and oxygenation for the first week (day 2–8); laboratory exams; chest x-ray examinations; need for intubation or ICU admission; treatment limitation decisions; and hospital outcome. The proportion of missing data for each variable is reported in Additional File 1: Table S1. An FiO_2_ of 90% was considered for PaO_2_/FiO_2_ ratio calculation during non-rebreathing oxygen mask therapy [24], albeit this estimation might lack of precision, particularly at higher values of minute ventilation [25]. Helmet CPAP failure was defined as a composite outcome of death or ICU admission for intubation. Albeit criteria for DNI decision or intubation were not protocolized, the MET was composed by a relatively small number of clinicians who shared decisions whenever possible and who met periodically to re-evaluate clinical strategies, decisions taken, controversies with colleagues, hence behaving consistently.

Safety of helmet CPAP treatment was evaluated by the presence of major adverse events, as recorded by the MET on the electronic medical records. Acute respiratory distress syndrome (ARDS) was defined following the Berlin definition [26].

### Statistical analysis

Statistical analysis was performed using SPSS 17.0 (SPSS Inc). Data are reported as means ± standard deviation (SD) or median [25–75 percentiles]. Continuous variables were tested for normality by Shapiro–Wilk test. Comparisons between patients’ groups were performed by Mann–Whitney or independent sample t-Test; comparisons within the same patient were performed by Wilcoxon or paired-sample t-Test, as appropriate. Comparisons between two categorical variables were performed by Fisher’s exact test (two-by-two comparisons) or by Chi-Square test (multiple classes). The analysis of helmet CPAP effects over PaO_2_/FiO_2_ ratio was conducted by repeated measures ANOVA, considering the PaO_2_/FiO_2_ before and after CPAP as within-subjects variables, and severity of impairment in gas exchange or treatment failure as between-subjects variable. The multivariate analyses to identify independent factors associated with failure were performed by backward logistic regressions, considering CPAP failure as the dependent dichotomous variable. We analyzed the treatment failure using the Kaplan–Meier approach with stratification for PaO_2_/FiO_2_ ratio assuming that patients discharged alive from hospital before 28 days were alive on that day; differences in time curves were assessed by the Log-Rank test. The level of significance was 0.05 (two-tailed test) unless otherwise specified to account for multiple comparisons (Bonferroni correction).

## Results

We enrolled in the study 306 consecutive patients (of the nearly 1500 COVID-19 treated) who failed oxygen mask therapy and underwent helmet CPAP treatment outside the ICU. Nearly 50% of the patients were younger than 65 years old and PaO_2_/FiO_2_ ratio with standard oxygen therapy was lower than 150 in two-thirds of the patients (209/306). The majority of the patients had no (30%) or one (30%) comorbidity; about half of the enrolled patients had hypertension (Table 1).

**Table 1.** Characteristics of study patients, comparing successful helmet CPAP treatment vs. failure

	All patients N = 306	Successful helmet CPAP treatment N = 159	Helmet CPAP failure N = 147	*P* value
Age, years	67 [58–76]	62 [54–70]	71 [63–79]	< 0.001*
Sex male, *n*. (%)	236 (77)	121 (76)	115 (78)	0.685
Body mass index, kg/m^2^	26 [24–30]	26 [24–30]	25 [24–30]	0.631
Any comorbidity, *n*. (%)	228 (74)	99 (62)	129 (88)	< 0.001*
Hypertension, *n*. (%)	159 (52)	69 (43)	90 (61)	0.002*
Comorbidities, *n*	1 [0–2]	1 [0–2]	2 [1–3]	< 0.001*
Clinical Frailty Scale	3 [2–4]	2 [2, 3]	3 [2–5]	< 0.001*
Symptoms onset to hospital admission, days	7 [4–10]	7 [5–10]	7 [4–10]	0.464
Hospital admission to oxygen therapy failure, days	1 [0–2]	1 [0–3]	0 [0–2]	0.001*
Do not intubate (DNI) order, *n* (%)	130 (42)	37 (23)	93 (63)	< 0.001*
White blood cells, n*10^3^/µL	7.38 [5.58–10.11]	7.03 [5.65–8.4]	7.71 [5.35–11.28]	0.052
Platelets, n*10^3^/µL	202 [153–260]	213 [164–265]	183 [142–256]	0.027
C-reactive protein, mg/L	109 [50–172]	86 [39–131]	144 [67–207]	< 0.001*
Procalcitonin, ng/mL	0.29 [0.13–1.05]	0.2 [0.11–0.63]	0.44 [0.2–1.42]	0.001*
Lactate dehydrogenase, U/L	420 [332–524]	369 [313–477]	475 [375–589]	< 0.001*
Creatinine, mg/dL	1 [0.8–1.3]	1 [0.8–1.1]	1.1 [0.9–1.5]	< 0.001*
Urea, mg/dL	39 [28–61]	32 [25–44]	50 [33–84]	< 0.001*

Univariate analysis of the association of relevant characteristics with CPAP failure. Data regarding the study population (All patients), the subgroup of patients successfully treated by helmet CPAP (Successful helmet CPAP treatment) and the subgroup of patients failing the helmet CPAP treatment (either intubated or non-survivors, depending on their ceiling-of-care status) are reported in the table

*Indicates persistence of statistically significant differences after Bonferroni correction for multiple comparisons (*p* < 0.003).

### Helmet CPAP treatment

**Fig. 1:**
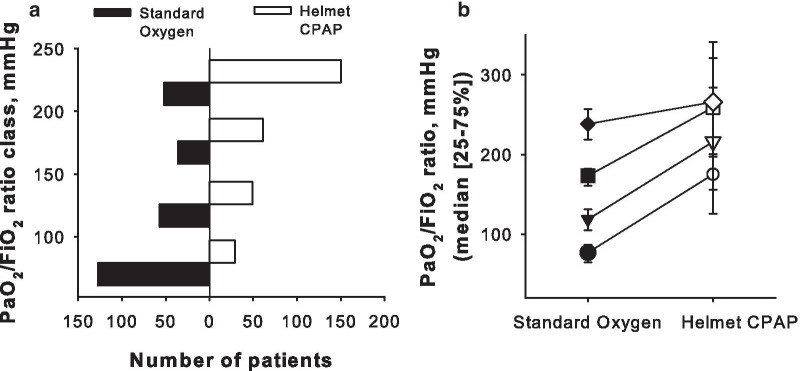
PaO_2_/FiO_2_ ratio increase with helmet CPAP therapy. The number of patients showing severe and moderate-to-severe gas exchange impairment is reduced shortly after start of helmet CPAP therapy (*P* < 0.001 by Chi-Square, panel a). PaO_2_/FiO_2_ ratio increase was higher among patients showing a more severe gas exchange impairment (*P* < 0.001 for helmet CPAP effect and for its interaction with severity class by RM Anova, panel b)

**Fig. 2:**
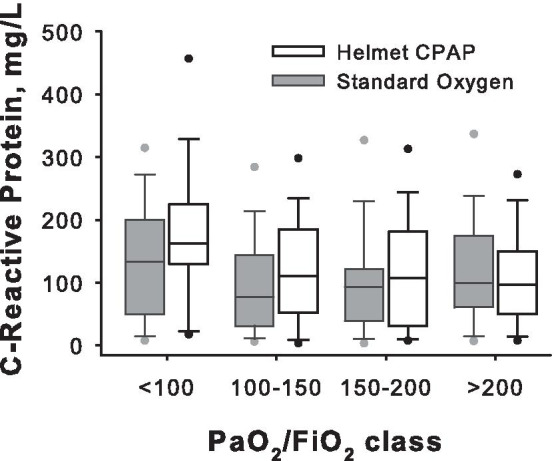
C-reactive protein levels and gas exchange impairment severity. C-reactive protein levels were not associated with gas exchange impairment severity during standard oxygen therapy (grey boxes, *P* = 0.088 by Anova). Patients showing a persistently severe gas exchange impairment despite helmet CPAP treatment (i.e., non-responders), showed higher levels of C-reactive proteins (white boxes, *P* = 0.009 by Anova). Boxes represent median and 25–75 percentiles; filled circles represent 5^th^ and 95^th^ percentiles

After failure of standard oxygen therapy, helmet CPAP treatment was started with a median PEEP of 5 [5–10] cmH_2_O and FiO_2_ of 50% [50–90]. Helmet CPAP therapy led to a dramatic oxygenation improvement: PaO_2_/FiO_2_ ratio doubled from about 100 to 200 mmHg (*P* < 0.001, Table 2). The median delay between the two arterial blood gases samplings (with standard oxygen mask and helmet CPAP) was rather short (median 3.5 [2–6] h), albeit we did not collect the exact timing at which CPAP was initiated between these two. The incidence of severe gas exchange impairment was markedly reduced by helmet CPAP (Fig. 1a, *P* < 0.001 by Chi-Square); the PaO_2_/FiO_2_ ratio improvement was more pronounced among patients who showed a more pronounced gas exchange impairment (Fig. 1b, *P* < 0.001 for helmet CPAP effect and for its interaction with PaO_2_/FiO_2_ class by RM Anova). A clinically significant reduction of respiratory rate was also present (from 28 [22–32] to 24 [20–29] BPM, *P* < 0.001). After beginning of helmet CPAP, 71% of patients presented ARDS criteria (severe 9%; moderate 35%; mild 27%). Higher levels of C-reactive proteins were detected in patients whose gas exchange impairment was not improved by helmet CPAP treatment (*P* = 0.009 by Anova, Fig. 2). Considering the entire population, helmet CPAP was maintained for 6 [3–9] days with a median PEEP of 10 [7–10] cmH_2_O and an FiO_2_ of 65 [50–90] %. During the first two days after start of CPAP therapy, helmet was maintained in place for an average of 21 h/day, from days three to five for an average of 19 h/day. Patients could eat and drink during the CPAP breaks. After the initial oxygenation improvement with helmet CPAP therapy, PaO_2_/FiO_2_ ratio remained steadily impaired during the first week (202 [128–284] mmHg).

**Table 2:** Respiratory parameters collected with standard oxygen therapy and shortly after start of helmet CPAP

	Standard oxygen N = 306	Helmet CPAP N = 306	*P* value
PaO_2_/FiO_2_ ratio, mmHg	103 [79–176]	202 [146–284]	< 0.001*
O_2_ saturation, %	95 [91–97]	98 [96–99]	< 0.001*
PaCO_2_, mmHg	33 [30–36]	33 [30.5–36]	0.011*
Resp rate, BPM	28 [22–32]	24 [20–29]	< 0.001*

BPM: breaths per minute; PEEP: positive end expiratory pressure; CPAP: continuous positive airway pressure; ARDS: acute respiratory distress syndrome

*Indicates persistence of statistically significant differences after Bonferroni correction for multiple comparisons (*p* < 0.012)

**Fig. 3:**
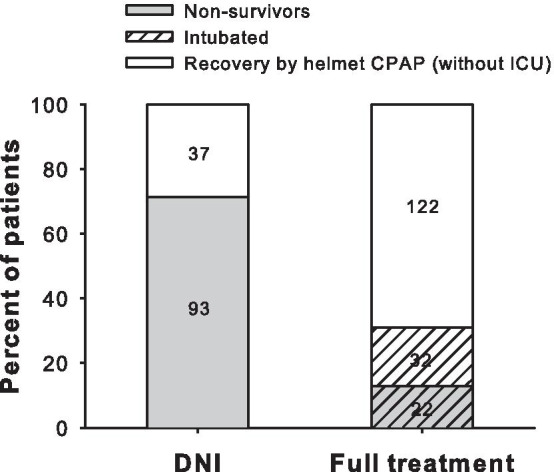
Treatment outcome among patients with or without limitations of care. Helmet CPAP therapy resulted effective in the majority of patients without any limitation of care (Full treatment). Helmet CPAP represented a valuable rescue therapy for patients who had failed standard oxygen therapy and had a Do Not Intubate (DNI) order

**Fig. 4:**
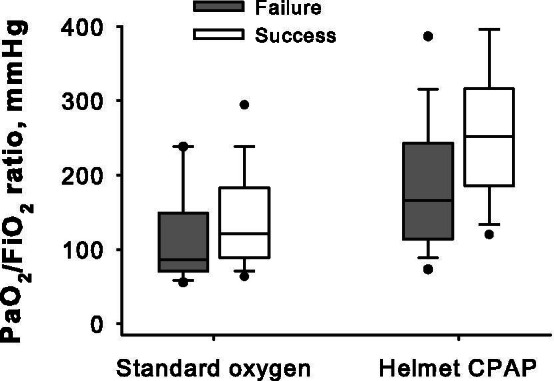
oxygenation improvement with helmet CPAP therapy. PaO_2_/FiO_2_ ratio increased with helmet CPAP therapy both in successful (Hospital discharge) and failure (intubated or non survivors) patients (*P* < 0.001 for CPAP effect). Patients in the successful treatment group showed a higher oxygenation response to CPAP (*P* = 0.002 for interaction between CPAP effect and Outcome by RM Anova)

**Fig. 5:**
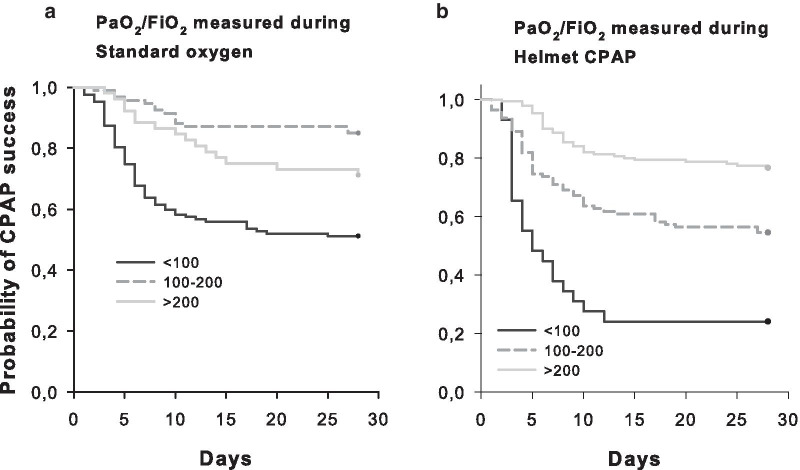
Helmet CPAP failure at 28 days. Probability of helmet CPAP failure at 28 days is presented stratifying the same population of patients depending on PaO_2_/FiO_2_ ratio measured either during standard oxygen (panel a) or during helmet CPAP (panel b). While a PaO_2_/FiO_2_ ratio below 100 mmHg with standard oxygen therapy is a weak predictor of failure (50% probability), showing the same oxygenation impairment during helmet CPAP is associated with a high probability of failure (80%, *P* < 0.001 by Log Rank for both)

Helmet CPAP failure occurred in 48% of the patients, mostly in patients who had a treatment limitation decision (72% vs. 31% in the full treatment group, *P* < 0.001, Fig. 3). CPAP failure was associated with several preexisting factors, such as advanced age, number of comorbidities and patient’s frailty, a data which was missing for 120 patients (Table 1). CPAP failure was strongly associated with worse gas exchange (Table 3, Fig. 4), increased inflammatory markers, higher levels of serum lactate dehydrogenase and worse renal function (Table 1). Successful treatment with CPAP (i.e., hospital discharge without intubation) was associated with a nearly double oxygenation response to helmet CPAP therapy as compared to failure (PaO_2_/FiO_2_ increase + 96 vs. + 53 mmHg, *P* = 0.001). Helmet CPAP failure probability showed a different pattern if patients were stratified according to PaO_2_/FiO_2_ ratio measured either during standard oxygen or during helmet CPAP (Fig. 5). A PaO_2_/FiO_2_ increasing above 200 mmHg after positioning helmet CPAP (68% in the successful vs. 32% in the failure groups, *P* < 0.001 by Chi-Square; *P* < 0.001 by Log Rank, Fig. 5); and a respiratory rate returning to clinically acceptable levels (22 vs. 28 BPM, *P* = 0.007, Table 2) were associated with successful helmet CPAP treatment.

**Table 3:** Respiratory parameters collected shortly after start of helmet CPAP in success vs failures

	All patients helmet CPAP *N* = 306	Successful helmet CPAP treatment *N* = 159	Helmet CPAP failure *N* = 147	*P* value
PaO_2_/FiO_2_ ratio, mmHg	202 [146–284]	252 [186–316]	166 [114–243]	< 0.001*
PaCO_2_, mmHg	33 [30.5–36]	33.2 [31–36.2]	32.5 [30–35.2]	0.091
Resp rate, BPM	24 [20–29]	22 [19–25]	28 [20–32]	0.007*
PEEP, cm H_2_O	5 [5–10]	5 [5–8]	5 [5–10]	0.010*
PaO_2_/FiO_2_ ratio increase with CPAP, mmHg	83 [28–154]	96 [45–176]	53 [18–115]	< 0.001*
ARDS diagnosis, *n* (%)	178 (71)	78 (62)	100 (79)	0.006*

Univariate analysis of the association of respiratory parameters collected shortly after CPAP start with CPAP failure during hospital stay

BPM: breaths per minute; PEEP: positive end expiratory pressure; CPAP: continuous positive airway pressure; ARDS: acute respiratory distress syndrome. Asterisks (*) indicates statistically significant differences after Bonferroni correction for multiple comparisons (*p* < 0.009)

A subgroup of patients (*n* = 42) was re-evaluated by the MET, on the first day of helmet CPAP therapy a few hours after initiation. At the receiving operator curve (ROC) analysis, the respiratory rate after few hours of helmet CPAP therapy was closely associated with CPAP success (AUC = 0.802 [95% CI = 0.66–0.94], *P* = 0.001). A respiratory rate below 30 BPM showed 100% sensitivity for CPAP success; a respiratory rate above 24 showed 81% sensitivity and 76% specificity for CPAP failure.

The adoption of prone position sessions during helmet CPAP treatment was frequent (45%).

No major adverse event associated with the use of helmet CPAP (e.g., deaths of full treatment patients before intubation) was recorded by MET during the study period.

### Full treatment patients

The majority of full treatment patients (122, 69%) did not require intubation and were successfully treated by helmet CPAP outside the ICU for 6 [4–9] days, with a PEEP of 10 [5–10] cmH_2_O and a FiO_2_ of 50 [35–80] %. Such patients were discharged from hospital after 14 [10–19] days. Patients requiring intubation (54, 31%) showed a higher heart rate on day one than those who did not (119, 68%) (92 [83–102] BPM vs. 80 [72–90], *P* < 0.001). They were transferred to ICU for intubation after 4 [3–7] days of helmet CPAP treatment; in those patients, prone positioning was almost always attempted before intubation.

While PaO_2_/FiO_2_ ratio during standard oxygen therapy did not differ between the success and failure groups (*P* = 0.280), helmet CPAP therapy led to a higher PaO_2_/FiO_2_ ratio in the success as compared to the failure group at the first measurement (257 [193–314] vs. 193 [133–259] *P* < 0.001); this corresponded to a more pronounced response in oxygenation (PaO_2_/FiO_2_ increase + 100 [45–162] mmHg vs. + 51 [12–99], *P* = 0.003). Among full treatment patients, a PaO_2_/FiO_2_ constantly above 150 mmHg during the first week was associated with a probability of recovery without intubation of 91% (*P* < 0.001 by Fisher’s exact test). Hospital mortality among full treatment patients was 12.5% (22/176). All deaths occurred after ICU admission; patients who eventually died spent a shorter period on CPAP as compared to ICU survivors (4 [2–5] vs. 5 [4–8] days, *p* = 0.05), probably due to a more severe and aggressive disease.

### DNI patients

A relevant number of patients (42%) had a treatment limitation decision (DNI). A DNI order was associated with age higher than 75 years old, a higher number of comorbidities and worse oxygenation both before and after helmet CPAP as compared to the full treatment group (*P* < 0.001 for all). A DNI order was strongly associated with helmet CPAP failure (which corresponds to mortality; *P* < 0.001). However, more than a quarter of DNI patients (28%) had a favorable outcome with helmet CPAP treatment outside the ICU, despite a relevant oxygenation impairment on day one both with standard oxygen and with helmet CPAP therapy (PaO_2_/FiO_2_ ratio 104 [81–180] and 224 [151–319], respectively; *P* < 0.001 for difference). Successful treatment among DNI patients was associated with younger age (*P* = 0.023) and lower comorbidities (*P* = 0.03).

### Multivariate analysis

Factors included in the model, to predict CPAP failure were: age, sex, number of comorbidities, C-reactive protein, body temperature on day one, time to oxygen mask failure, PaO_2_/FiO_2_ ratio and PaCO_2_ both during standard oxygen and at the first measurement during helmet CPAP. At the backward logistic regression analysis, C-reactive protein, time to oxygen mask failure, age, PaO_2_/FiO_2_ ratio collected during helmet CPAP treatment and number of comorbidities were independently associated with helmet CPAP failure (Table 4). The other tested factors (sex, body temperature, PaO_2_/FiO_2_ ratio and PaCO_2_ during standard oxygen treatment, PaCO_2_ measured during CPAP) did not emerge as independent predictors of failure.

**Table 4:** Multivariate analysis of factors associated with helmet CPAP failure

Factor^a^	*P* value	Odds ratio [95% C.I.]
C-reactive protein, mg/L	0.001	1.006 [1.003–1.010]
Hospital admission to oxygen therapy failure, days	0.001	0.775 [0.661–0.908]
Age, years	0.002	1.054 [1.020–1.089]
PaO_2_/FiO_2_ ratio (helmet CPAP), mmHg	0.003	0.995 [0.991–0.998]
Comorbidities, *n*	0.005	1.582 [1.147–2.182]

CPAP: continuous positive airway pressure

^a^factors entered in the backward regression model and removed due to lack of statistical significance (*P* > 0.05): sex, body temperature, PaO_2_/FiO_2_ ratio and PaCO_2_ during standard oxygen treatment, PaCO_2_ measured during CPAP. C-reactive protein, time to oxygen mask failure, age, PaO_2_/FiO_2_ ratio collected during helmet CPAP treatment and number of comorbidities were independently associated with helmet CPAP failure.

The list of factors entered in the backward analysis is reported in Additional File 1: Table S2. The results of a similar multivariate analysis including the PaO_2_/FiO_2_ ratio change due to helmet CPAP in place of the PaO_2_/FiO_2_ ratio measured during helmet CPAP are reported in Additional File 1: Table S3.

## Discussion

In this observational study, we present the outcomes of helmet CPAP therapy for acute respiratory failure during the COVID-19 pandemic in a large Italian center. Helmet CPAP therapy outside ICU was feasible for several days (approximately one week), despite a severe gas exchange impairment. It was safely used to improve oxygenation and reduce respiratory distress (indirectly assessed, in a subset of patients, by respiratory rate) on the hospital floor, representing an intermediate-level therapy to prevent intubation or ICU admission in nearly 70% of the full treatment patients. In DNI patients who failed standard oxygen therapy, a rescue trial with helmet CPAP represented a last treatment option with low invasiveness, which might have contributed to the observed 30% survival.

In our hospitals, we have been using helmet CPAP since several years to apply PEEP during respiratory failure outside the ICU. Our approach to COVID-19 pneumonia, as in other types of pneumonia before the outbreak, is based on the rationale that PEEP and FiO_2_ are the cornerstones of respiratory support when non-rebreathing oxygen mask fails and can be delivered noninvasively, safely and effectively on the hospital floor by helmet CPAP. Therefore, helmet CPAP represented a natural and valuable choice to face the pandemic and limit the need for intubation.

A free-flow venturi system is simple to set up and operate; a refresh course was provided to instruct floor personnel, allowing us to rapidly escalate the treatment to hundreds of hypoxemic patients. In comparison with “classical” pressure support, the use of free flow helmet CPAP has the main advantages of not requiring a ventilator (whose shortage represented a major issue during pandemics) and not presenting any issue of patient–ventilator synchrony, even in the presence of leaks, elevated respiratory rate, patient movement and so forth. Taking advantage of a nurse-managed optimization bundle, helmet CPAP was well tolerated for many hours a day (almost continuously during the first days) [14], without relevant pressure ulcers [22].

All the enrolled patients were in the need of upscale treatment, either for hypoxemia or respiratory distress; however, it was crucial to carefully select and minimize the number of patients undergoing intubation and to have a safe bridge for those in whom intubation had to be postponed for logistical reasons. Helmet CPAP led to a marked and persistent oxygenation improvement in both full treatment and DNI patients, possibly indicating lung recruitment [27]. Ventilator effort was also reduced, as shown by lower respiratory rate shifting toward normal values after start of helmet CPAP therapy. Unfortunately, the way in which clinicians measured respiratory rate was not specified a priori, standardized or even recorded and might be affected by several limitations [28]. Hence, helmet CPAP might have reduced the so-called patient self-inflicted lung injury, whose putative existence and clinical is a matter of debate [9, 29]. In this respect it must be also noted that a limitation inherent to the use of helmet is the inability of monitoring tidal volume, an important parameter to predict NIV failure [30].

The criteria to define standard oxygen therapy failure were quite conservative, leading to an early delivery of PEEP to hypoxemic patients. We cannot exclude that few patients could have been treated with non-rebreathing mask for longer periods of time; however, the persistent oxygenation impairment over the study days (median PaO_2_/FiO_2_ ratio below 200) and the high FiO_2_ need after start of helmet CPAP suggest worsening conditions, which would necessarily lead to escalate therapy.

The vast majority of full treatment patients (about 70%) was successfully treated with helmet CPAP without escalating to intubation, suggesting that a prolonged helmet CPAP treatment is effective for COVID-19 respiratory failure with a 24/7 availability of the MET. A PaO_2_/FiO_2_ ratio above 150 mmHg during helmet therapy was associated with a positive predictive value of 91% for treatment success. At the multivariate analysis, a lower PaO_2_/FiO_2_ value measured shortly after start of helmet CPAP was associated with failure, independent from age. Taken together, such data suggest that a helmet CPAP trial might provide useful information to the clinician about the evolution of the respiratory failure: simple markers such as a clear oxygenation improvement shortly after start of CPAP, a respiratory rate falling below 24 BPM within few hours, a PaO_2_/FiO_2_ persistently above 150 during the days, indicate that the patient can be treated effectively by helmet CPAP and possibly outside the ICU. In a previous trial, Patel’s et al. enrolled full treatment patients needing CPAP and compared helmet with face mask, showing a much lower need for intubation with helmets (18% vs. 61%) [10]. The 31% failure rate recorded in our population is slightly higher than Patel’s trial; possible explanations for the higher failure recorded in our population may be 1) the lack of a proven therapy for COVID-19 patients (either etiological or supportive, since steroids were not consistently used in March, 2020) and 2) the inability of replicating in the hospital ward the typical ICU tight monitoring for a vast number of patients.

The benefits of helmet CPAP therapy were evident also in the DNI group, where this strategy might have contributed to the 30% survival of DNI patients, who had no other treatment option for respiratory failure. The older age and the higher number of comorbidities suggest that preexisting conditions were the major culprits for failure in the DNI group, limiting the benefits of therapies focused on respiratory support such as helmet CPAP.

We acknowledge that we cannot draw definite conclusions about the timing and the effectiveness of CPAP therapy due to the observational nature of our data. However, a randomized trial comparing the use of CPAP vs. early intubation was not feasible during the pandemic for the shortage of ICU beds and might be considered unethical under different perspectives, due to the different invasiveness and risks for patients treated by CPAP as compared to intubation.

A different and widely used option to treat hypoxemic patients unresponsive to non-rebreathing oxygen mask are high flow nasal cannula (HFNC) [31]. We chose helmet CPAP as noninvasive respiratory support device for several reasons. First, HFNC provide a PEEP level much lower than CPAP, possibly representing a “low-dose” therapy for patients affected by severe gas exchange impairment. Second, the need for a dedicated heating and humidifying system with HFNC limited the use on a restricted number of patients, while active humidification may not be mandatory when spontaneously breathing a mixture of medical (dry) and ambient gas as within a Venturi based helmet CPAP. Third, the use of HFNC presented concerns for staff and environment contamination due to droplet spread, while helmet CPAP was the ideal device to limit droplet diffusion when using a HEPA filter on the outlet gas port [14]. Lastly, the MET and floor staff were already familiar with helmet CPAP, which has been used outside the ICU for many years in our hospital.

A limitation of our approach is therefore that the failure rate might not be the same if this technique was applied in other contexts, with difference experience, protocols or patients selection. Another limitation is that data were collected during a specific pandemic: Adherence to hospital protocols was more difficult due to the increased clinical burden; some patients may have received intubation later than usual due to ICU bed shortage; DNI orders may have been used more often than usual, denying ICU trials in elderly patients; data about patient comfort with the selected CPAP interface were not collected. Finally, the threshold and predictive values which we report (e.g., for PaO2/FiO2 and respiratory rate) were not evaluated prospectively. All the presented factors may limit the generalizability of our data to patients affected by respiratory failure due to other etiologies. On the other side, the need to treat such a huge number of respiratory failure patients outside the ICU proved that helmet CPAP is a feasible and effective choice.

## Conclusions

We showed that treatment of acute respiratory failure patients outside the ICU is feasible with helmet CPAP for many days, despite a persistent relevant degree of gas exchange impairment. Treatment was also effective, leading to a marked oxygenation improvement; helmet CPAP therapy was associated with a good outcome in the vast majority of full treatment patients and an effective rescue for a limited but significant proportion of DNI patients.

## Supplementary Information

**Additional file 1**. A PDF (portable document format) file containing two figures (e-Figure 1, 2) and three tables (e-Table 1, 2 and 3).

## Data Availability

The datasets used and analyzed during the current study are available from the corresponding author after obtaining the approval of the Spallanzani Institute ethical committee.

## Abbreviations

ARDS: Acute respiratory distress syndrome
BPM: Breaths per minute
COVID-19: Novel coronavirus disease
CPAP: Continuous positive airways pressure
DNI: Do not intubate
HFNC: High flow nasal cannula
ICU: Intensive care unit
MET: Medical emergency team
PEEP: Positive end expiratory pressure
