# Supplementary material for: Helmet CPAP to treat hypoxic pneumonia outside the ICU: an observational study during the COVID-19 outbreak
Source: Crit Care. 2021 Feb 24;25:80. doi: 10.1186/s13054-021-03502-y (PMC7903369; doi:10.1186/s13054-021-03502-y)
Supplement: Supplementary file 1 — Additional file 1. A PDF (portable document format) file containing two figures (e-Figure 1, 2) and three tables (e-Table 1, 2 and 3). [file 13054_2021_3502_MOESM1_ESM.pdf]

Additional File 1

Helmet CPAP to treat hypoxic pneumonia outside the ICU: an observational study during the COVID-19 outbreak

Andrea Coppadoro[1] M.D., Annalisa Benini[1] M.D., Robert Fruscio[1,2] M.D., Luisa Verga[1] M.D., Paolo Mazzola[1,2] M.D., Giuseppe Bellelli[1,2] M.D., Marco Carbone[1,2] M.D., Giacomo Mulinacci[1,2] M.D., Alessandro Soria[1] M.D., Beatrice Noè[1,2] M.D., Eduardo Beck[3] M.D., Riccardo Di Sciacca[1] M.D., Davide Ippolito[1] M.D., Giuseppe Citerio[1,2] M.D., Maria Grazia Valsecchi[2], Andrea Biondi[1,2] M.D., Alberto Pesci[1,2] M.D., Paolo Bonfanti[1,2] M.D., Davide Gaudesi[2], PhD, Giacomo Bellani[1,2]* M.D. Ph.D., Giuseppe Foti[1,2] M.D.*

* These authors contributed equally to the manuscript

[1]ASST Monza, San Gerardo Hospital, Monza, Italy

[2] Department of Medicine and Surgery, University of Milan-Bicocca, Monza, Italy

[3] ASST Monza, Desio Hospital, Desio, Italy

Corresponding Author

Giacomo Bellani, MD, PhD

University of Milan-Bicocca, Department of Medicine and Surgery

Via Cadore 48, Monza (MB), Italy

giacomo.bellani1@unimib.it

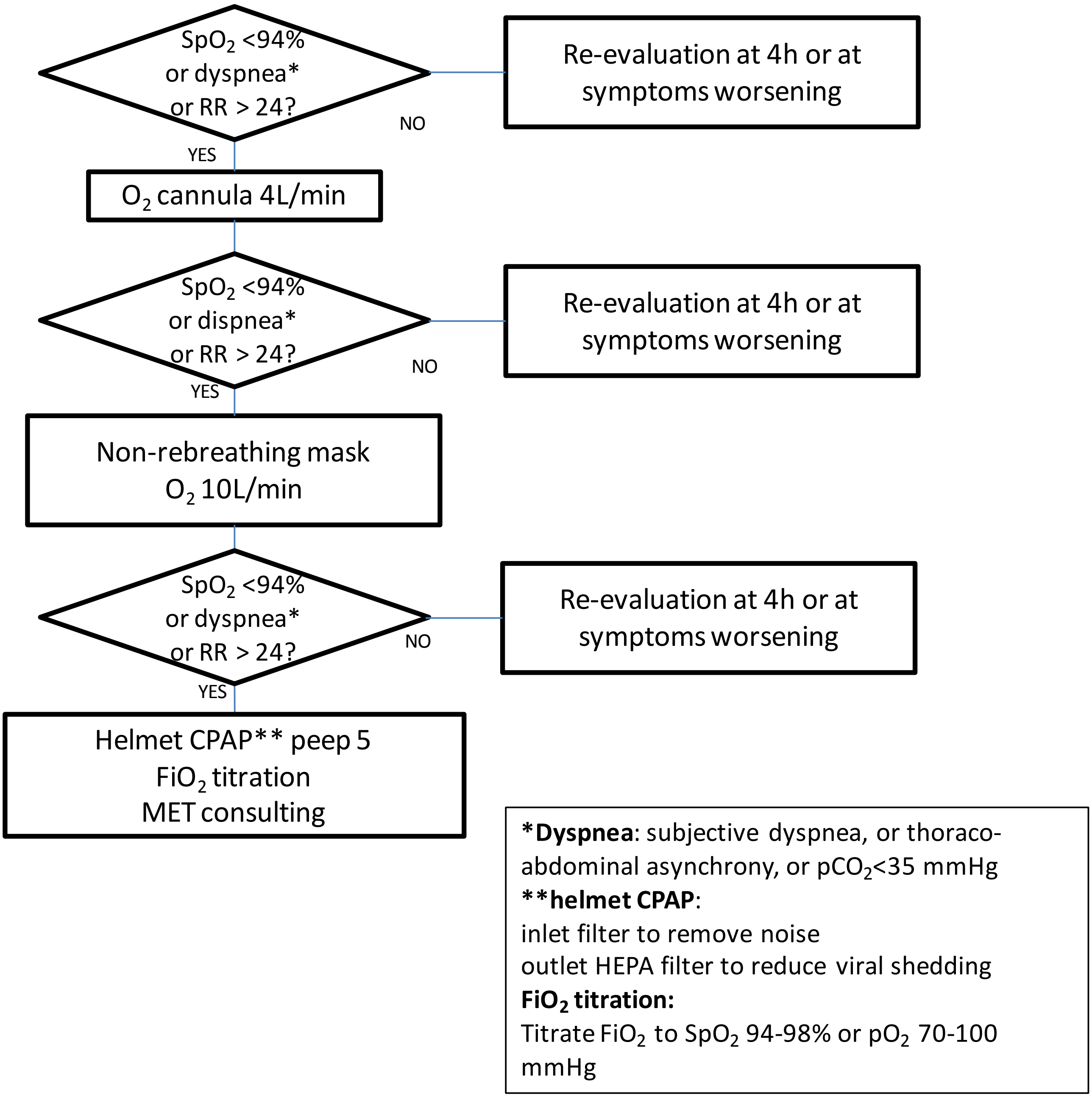

e-Figure 1. Simplified protocol for helmet CPAP therapy start. RR respiratory rate; MET medical emergency team; HEPA high efficiency particulate air

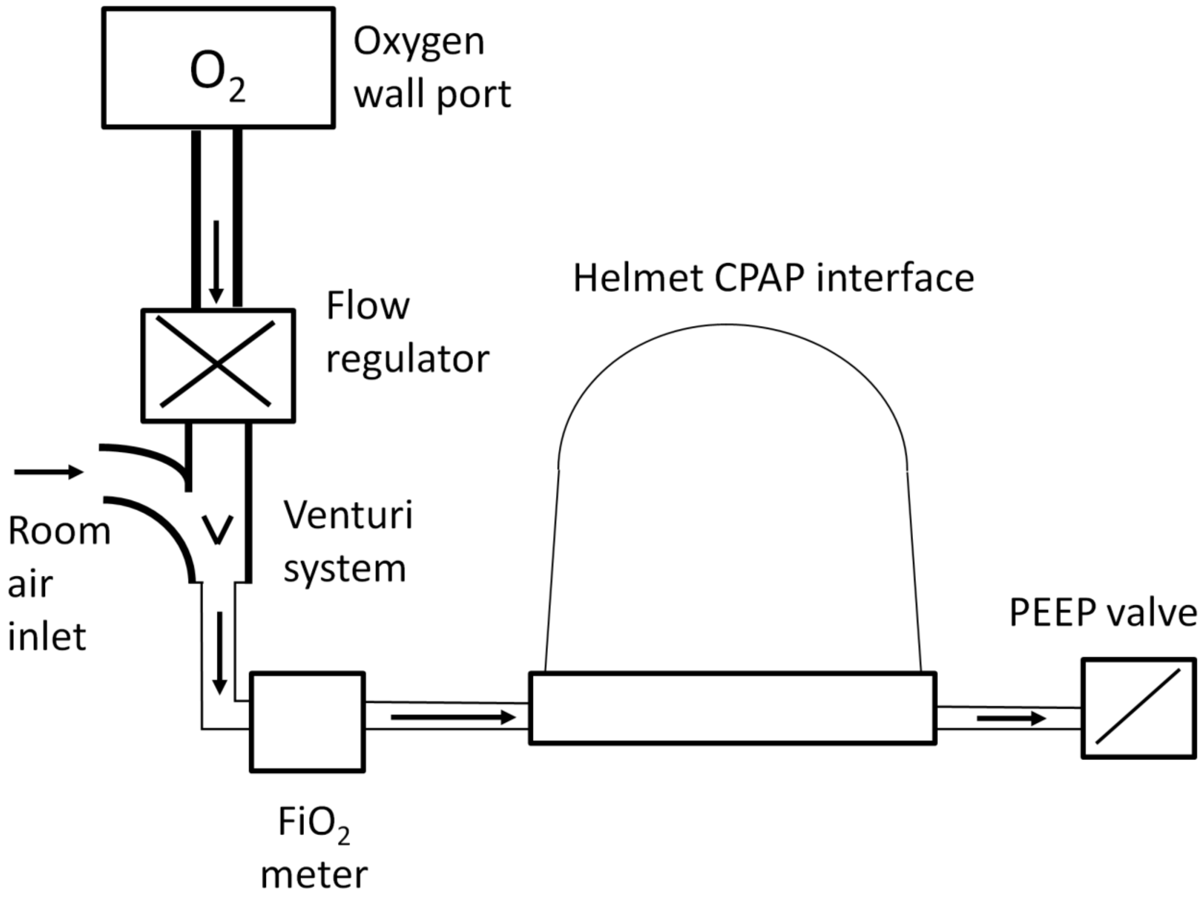

e-Figure 2. Scheme of helmet CPAP free flow circuit. Helmet CPAP is inflated by a gas mixture of medical Oxygen and room air generated by a Venturi system. Gas flow was usually set at 60-90L/min, based on patient distress, and FiO_2 was adjusted based on patient oxygen saturation (measured either by arterial blood gas or peripheral saturation). Pressure within the helmet was maintained constant (5-10 cmH_2O) by a PEEP valve placed on the helmet gas outlet. Placing a HEPA filter before PEEP valve (not shown in figure) is suggested to prevent viral shedding; to reduce noise within the helmet, HME filters (not shown) might be connected to the Venturi system and smooth (internal surface) tubing are recommended.

e-Table1. Number of missing data by study variable

Variable	Missing	Variable	Missing
Age	0	PaO2/FiO2 Standard O2	34
Sex	0	O2 sat Standard O2	53
Body mass index	191	PaCO2 Standard O2	33
Comorbidities, N	0	Respiratory rate St.O2	167
Clinical Frailty Scale	120	PaO2/FiO2 CPAP	17
Symptoms to Hosp. time	6	O2 sat CPAP	29
Admission to CPAP time	1	PaCO2 CPAP	13
DNI	0	Respiratory rate CPAP	211
White blood cells	21	PEEP CPAP	7
Platelets	26	FiO2 CPAP	6
C-Reactive protein	21	ARDS	54
Procalcitonin	163	CPAP days	2
Lactate Dehydrogenase	103	Pronation	55
Creatinine	19	Body temperature	25
Urea	56	Pre/post CPAP ABG time delay	62

e-Table2. First step of the backward multivariate analysis of independent predictors of helmet CPAP failure

Factor[a]	*P* value	Odds Ratio [95% C.I.]
$PaCO_2$ (standard oxygen), mmHg	0.405	0.963 [0.881-1.053]
$PaCO_2$ (CPAP), mmHg	0.611	1.025 [0.932-1.126]
Body temperature, C^o	0.476	1.136 [0.800-1.615]
PaO_2/FiO_2 ratio (standard O_2), mmHg	0.313	0.997 [0.992-1.003]
Sex	0.385	0.696 [0.307-1.578]
CRP, mg/L	0.001	1.006 [1.003-1.010]
Hospital admission to oxygen therapy failure, days	0.006	0.791 [0.669-0.935]
Age, years	0.004	1.050 [1.016-1.085]
PaO_2/FiO_2 ratio (CPAP), mmHg	0.007	0.995 [0.991-0.999]
Comorbidities, n.	0.008	1.567 [1.127-2.179]

A p value of 0.05 was chosen as cut-off for factor removal.

e-Table3. Multivariate analysis of independent predictors of helmet CPAP failure

**Factor**[a]	***P* value**	**Odds Ratio [95% C.I.]**
CRP, mg/L	0.001	1.006 [1.003-1.010]
Hospital admission to oxygen therapy failure, days	0.001	0.767 [0.653-0.900]
Age, years	0.002	1.053 [1.019-1.088]
PaO_2/FiO_2 ratio (standard O_2), mmHg	0.007	0.993 [0.987-0.998]
PaO_2/FiO_2 ratio increase by CPAP, mmHg	0.008	0.995 [0.991-0.999]
Comorbidities, n.	0.009	1.544 [1.115-2.137]

[a] factors entered in the backward regression model and removed due to lack of statistical significance (P>0.05): sex, body temperature, PaO_2/FiO_2 ratio and $PaCO_2$ during standard oxygen treatment, $PaCO_2$ measured during CPAP
